# Risk factors analysis and the establishment of nomogram prediction model for PICC-related venous thrombosis in patients with lymphoma: a double-center cohort-based case-control study

**DOI:** 10.3389/fonc.2024.1347297

**Published:** 2024-03-15

**Authors:** Xue-xing Wang, Yuan He, Jie Chu, Jin-song Xu

**Affiliations:** ^1^Department of Oncology, Anning First People’s Hospital Affiliated to Kunming University of Science and Technology, Kunming, China; ^2^Department of Geriatric Oncology, The Third Affiliated Hospital of Kunming Medical University, Yunnan Cancer Hospital, Kunming, China; ^3^Department of Oncology, The First People’s Hospital of Ziyang, Ziyang, China

**Keywords:** nomogram, catheterization, prognosis, logistic models, lymphoma neoplasms

## Abstract

**Objective:**

The objective of this study is to examine the risk factors associated with the occurrence of PICC-Related Venous Thrombosis (PICC-RVTE) in individuals diagnosed with lymphoma, as well as to develop a predictive risk nomogram model.

**Methods:**

A total of 215 patients with lymphoma treated at Yunnan Provincial Tumor Hospital from January 2017 to December 2020 were retrospectively evaluated as the training cohort; 90 patients with lymphoma treated at the Department of Oncology of the First People’s Hospital of Anning, Affiliated to Kunming University of Science and Technology during the January 2021 to September 2023 were evaluated as the validation cohort. Independent influencing factors were analyzed by logistic regression, a nomogram was developed and validated, and the model was evaluated using internal and external data cohorts for validation.

**Results:**

A total of 305 lymphoma patients were selected and 35 (11.48%) PICC-RVTE occurred, the median time was 13 days. The incidence within 1-2week was 65.71%. Multivariate analysis suggested that the activity amount, thrombosis history(within the last 12 months), ATIII, Total cholesterol and D-dimer levels were independently associated with PICC-RVTE, and a nomogram was constructed based on the multivariate analysis. ROC analysis indicated good discrimination in the training set (area under the curve [AUC] = 0.907, 95%CI:0.850-0.964) and the testing set (AUC = 0.896, 95%CI: 0.782-1.000) for the PICC-RVTE nomogram. The calibration curves showed good calibration abilities, and the decision curves indicated the clinical usefulness of the prediction nomograms.

**Conclusions:**

Patients should be advised to undergo color Doppler ultrasound system testing within two week after the implantation of a PICC catheter to detect PICC-RVTE at an early stage. The validated nomogram can be used to predict the risk of catheter-related thrombosis (CRT) in patients with lymphoma who received at least one chemotherapy after PICC catheterization, no bleeding tendency, no recent history of anticoagulant exposure and no severe heart, lung, renal insufficiency. This model has the potential to assist clinicians in formulating individualized treatment strategies for each patient.

## 1 Introduction

In recent years, peripherally-inserted central catheters (PICCs) have gained widespread utilization in clinical practice due to their perceived safety, user-friendliness, extended duration of use, ease of maintenance, minimal discomfort, and reduced risk of infection (1, 2). Moreover, PICCs have emerged as a preferred alternative to central venous catheters (CVCs) among healthcare providers and patients alike, primarily due to their ability to effectively mitigate severe complications such as pneumothorax and gas embolism. Consequently, PICCs are progressively supplanting CVCs as the primary venous access route for oncology patients undergoing chemotherapy, blood transfusion, or nutritional support (3).

Despite the many advantages of PICC, PICC-related complications are still a problem that medical practitioners need to pay attention to in clinical practice, among which, the most common and noteworthy problem is still PICC-RVTE, which not only interrupts the treatment of oncology patients, but also increases the cost of treatment, prolongs the treatment time of patients, and even more seriously, leads to the death of patients. There is a considerable difference in the incidence rates of PICC-related venous thrombosis among studies conducted previously. However, considering the growing use of PICCs, verifying the exact incidence and risk of venous thrombosis associated with PICCs is important. Understanding the risk of venous thrombosis is crucial to patient safety as well.

Currently in numerous previous studies, most investigators focused on investigating and analyzing the risk factors associated with CVC-related veins and the analysis of predictive value in solid tumors (4, 5). Risk factors for PICC-RVTE in patients with non-solid tumors are unknown, as few studies have focused on this issue. In this study, we investigated useful predictors and developed a validated prediction model for PICC-RVTE in patients with lymphoma.

## 2 Methods

### 2.1 Study design and patients selected

**Figure 1:**
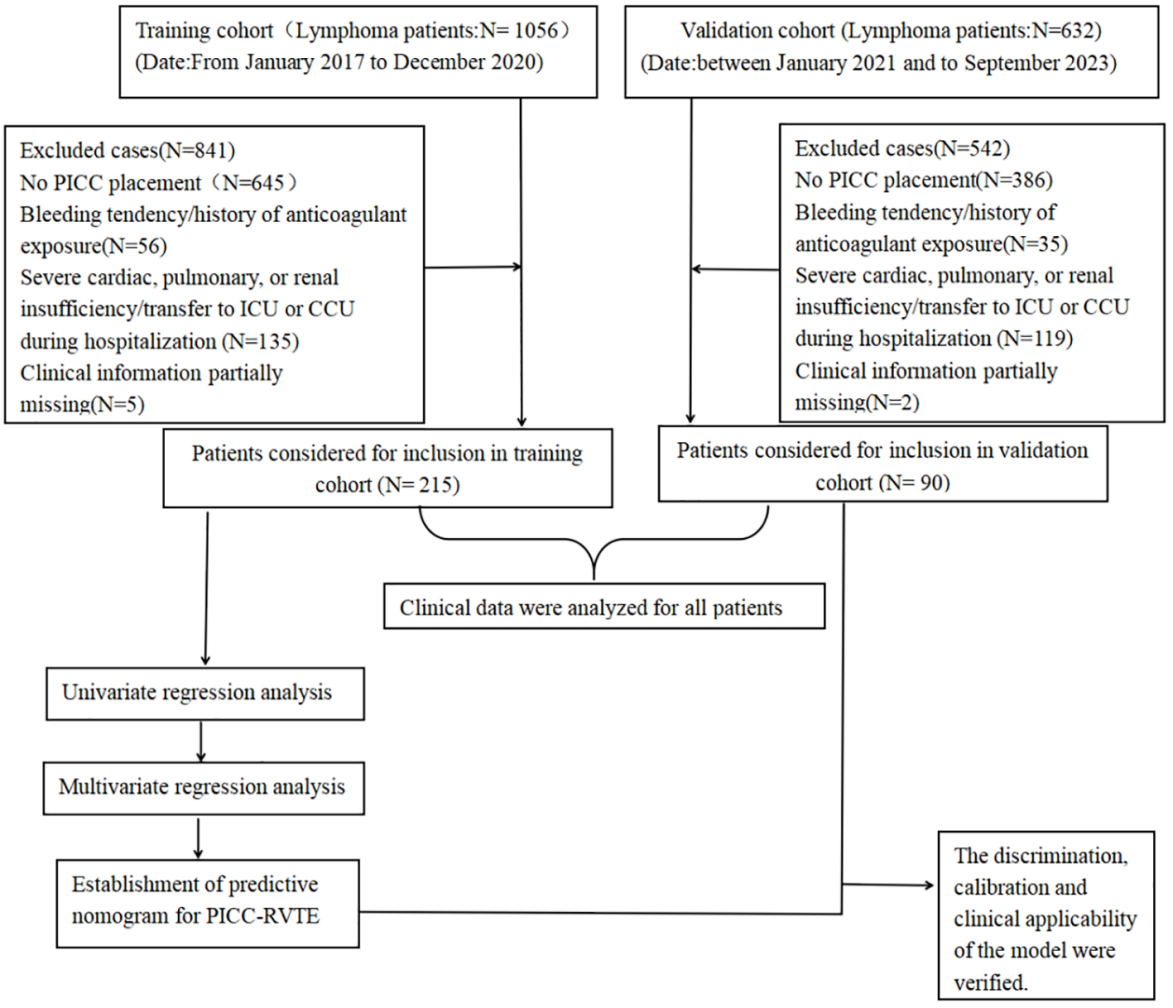
Experimental roadmap of this study. This flow diagram indicates the workflow of the method present in this study.

The medical records of 215 and 90 patients with lymphoma who underwent PICC insertion at the Third Affiliated Hospital of Kunming Medical University and the Anning First People’s Hospital Affiliated to Kunming University of Science and Technology between January 2021 and September 2023 were retrospectively reviewed. The inclusion criteria were as follows: 1) Patients with confirmed lymphoma based on histopathological, cytological, and imaging examinations. 2) Patients who underwent PICC placement through basilic vein, cephalic vein or cubital vein with an occurrence of thrombosis confirmed by ultrasound examination. 3) All participants in the study underwent intravenous angiography, non-enhanced ultrasound, or enhanced echocardiography to verify the presence of PICC-RVTE subsequent to PICC catheterization until the PICC removal or thrombosis occurred. 4) All patients received at least one cycle of chemotherapy. The exclusion criteria included: 1) patients with a bleeding tendency or history of exposure to anticoagulant drugs (long-term oral anticoagulant drugs or discontinuation of anticoagulant drugs for less than two weeks outside the hospital or in the hospital within two weeks; patients with Prothrombin Time - International Normalization Ratio (PT-INR) greater than 1.3 after warfarin treatment) 2) patients with severe heart, lung, renal insufficiency, or referral to ICU or CCU during hospitalization; 3) Patients diagnosed with solid malignancies. 4)A sample of cases lacked clinical data (≥20%) was excluded from the study (**Figure 1** shows an experimental roadmap). Because of the retrospective nature of the study, the study protocol was approved by the two Hospital Ethics Committee (The Third Affiliated Hospital of Kunming Medical University/Yunnan Cancer Hospital, Kunming, 650000 & The First People’s Hospital of Anning City, Kunming, 650300 (NO.KYLX2023-101 &NO.2023-018-01)),which waived the requirement for informed consent as the data were analyzed anonymously and the patient privacy was protected. This study was carried out in accordance with Good Clinical Practice (GCP) guidelines and the Declaration of Helsinki.

This experimental roadmap indicates the inclusion and exclusion for patients and the workflow of the method present in this study.

### 2.2 Data collection

The process of data collection and data review was conducted autonomously by two researchers who were trained at each of the two centers, resulting in a total of four individuals involved. The following detailed information of each patient was collected through the hospital information systems (HIS),including Clinical characteristics, medical history, clinical indicators, biochemical indicators and PICC catheter information.

The clinical characteristics of the patients mainly include: Age, gender, diagnosis, Karnofsky Performance Status (KPS) score, height, weight, body mass index (BMI), history of smoking, drinking, surgery, hormones usage, infection states during catheterization, hypertension, diabetes, hyperlipidemia, thrombosis, and hypercoagulable state. The definition of hypertension encompasses individuals with a systolic blood pressure (SBP) equal to or exceeding 140 mmHg, or a diastolic blood pressure (BP) equal to or exceeding 90 mmHg, who are presently undergoing treatment for hypertension or have been diagnosed with the condition. Similarly, the definition of diabetes includes individuals with a fasting glucose level equal to or exceeding 7.0 mmol/L, who are currently receiving treatment for diabetes or have previously been diagnosed with the condition. Hyperlipidemia was operationally defined as having a total cholesterol level exceeding 220 mg/dL or receiving medical intervention for the management of hyperlipidemia.

The laboratory indicators including blood routine (red blood cells, white blood cells, platelets, hemoglobin), coagulation mechanism (PT, INR, PT ratio, APTT, TT, FIB, plasma antithrombin III, fibrinogen degradation products, and D-dimer). The biochemical indicators including albumin, globulin, direct bilirubin, indirect bilirubin, triglyceride, total cholesterol, and fasting blood glucose. The examination of coagulation function was conducted utilizing a Sysmex cs-5100 automatic coagulometer and Siemens routine coagulation reagents sourced from SYSMEX (Japan). Hematocrit and biochemical analysis were carried out employing Sysmex xt-500i and Sysmex xt-2000i instruments. Among these, laboratory test indices were gathered one to two weeks before the occurrence of PICC-RVTE. The PICC catheter information including history of blood transfusion using PICC, history of CVC implantation, history of central venous catheterization, limb of catheterization, vein of catheterization, The location of the catheter tip, the length of the catheter, and the amount of activity of the catheterized limb.

### 2.3 Statistical analysis

The clinical trial data were collected using Excel 2019.SPSS 28.0 (IBM, Armonk, NY, USA) and R package (version 4.1.3) were used for statistical analysis. Categorical variables were quantified using numerical values and percentages, while continuous variables were represented as mean ± SD. Statistical disparities among categorical variables were assessed using either the χ^2^ test or Fisher’s exact test, whereas continuous variables were evaluated using two-tailed t-tests or Mann-Whitney U tests. The factors with statistical significance in univariate logistic regression analysis were further included in multivariate analysis, and related risk factors were analyzed by logistic regression model. Based on the results of multivariable logistic regression analysis, a nomogram was developed. The performance of the nomogram was assessed using the concordance index (C-index) by comparing the predicted probabilities of PICC-RVTE formation from the nomogram with the observed probabilities. The internal effectiveness of the model is evaluated by Bootstrapping. The same number of samples are randomly selected from the original data set to form a training set, and the unselected samples form a test set. This process is repeated 1000 times. A higher C-index indicated a more precise predictive ability. Calibration curves were generated, and the Hosmer Lemeshow test (HL test) P value was calculated to assess the model’s over-fitting performance. The clinical utility of the nomogram prediction was assessed using decision curve analysis (DCA). The predictive values of the PICC-RVTE risk nomogram model were compared using ROC analysis and the Delong test P value. In this study A *p-value* less than 0.05 was deemed to indicate statistical significance, and the testing was 2-sided.

### 2.4 Classification of PICC-RVTE

According to the extent of the involved vein PICC-RVTE was categorized into 2 types: superficial vein thrombosis (SVT) and deep vein thrombosis (DVT). Catheter-associated thrombosis can be categorized into 3 types based on the specific location of thrombus formation detected by ultrasound: (1) vascular adherent thrombus, which is thrombus attached to the target vein wall; (2) pericatheteric sheath, which is thrombus adhering only to the catheter surface; and (3) mixed thrombus, which is vein wall thrombus with catheter adherent cuff thrombus. PICC-RVTE can be classified as symptomatic thrombus and asymptomatic thrombus according to the presence or absence of clinical manifestations, and the signs or symptoms associated with PICC-RVTE include localized pain, infection, edema, dyspnea, and heart failure.

### 2.5 PICC insertion and maintenance

All PICC catheterizations had been operated on under ultrasound guidance by an experienced and qualified professional nurse. The catheters were maintained and handled by specialized PICC nurses using sterile techniques throughout the study. All PICCs used in this study were single-lumen catheters (PowerPICC, Bard Access Systems, Inc., Salt Lake City, Utah, USA) with a model of 4-French or 5Fr,and were routinely maintained by PICC specialist nurses using sterile technique weekly. A 45% catheter-to-vein ratio limit was used when inserting PICC devices. All the procedures were performed in accordance with the PICC specifications. PICC tip position at the cavoatrial junction is all confirmed via X-ray.

## 3 Results

### 3.1 Study population and characteristics

**Figure 2:**
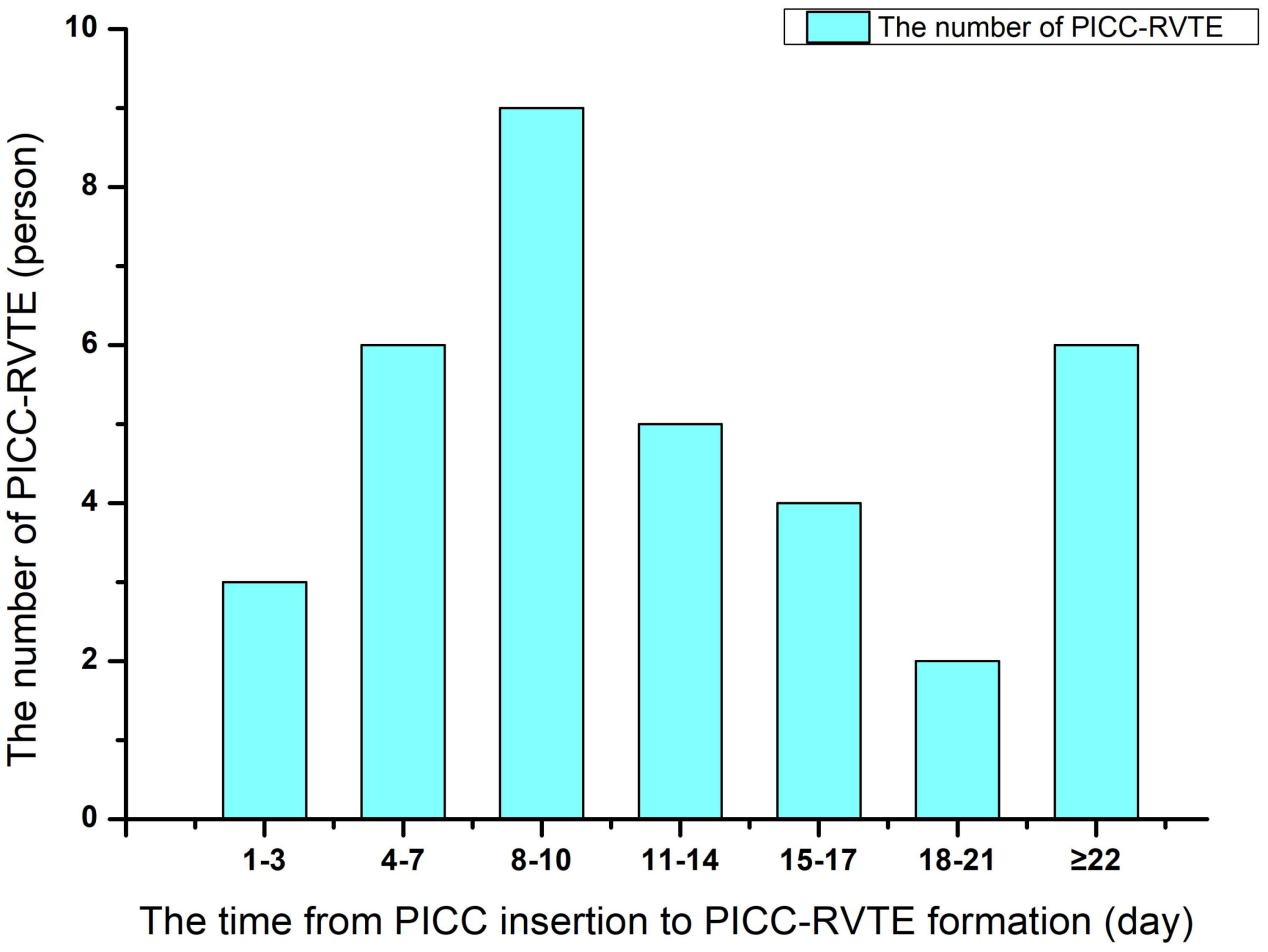
The time distribution between peripherally inserted central catheter (PICC) insertion to the onset of thrombosis.

Case data from 215 and 90 patients with lymphoma undergoing PICC implantation from the Third Affiliated Hospital of Kunming Medical University and the First People’s Hospital of Anning affiliated with Kunming University of Science and Technology were reviewed as the development set and validation set during the time periods of January 2021 to June 2022 and July 2022 to September 2023, respectively. Among all included patients with lymphoma,35 patients (11.48%) developed PICC-RVTE after PICC placement and were assigned to the thrombosis group, while the remaining 270 patients (88.52%) were assigned to the non-thrombosis group. The average duration from PICC insertion to the onset of thrombosis was found to be 13.00 days. A significant proportion of patients (65.71% (23/35)) experienced PICC-RVTE within 1-2weeks following PICC implantation. The complete time distribution of PICC-RVTE can be observed in **Figure 2**. On further analysis, the most common PICC-RVTE was vascular adherent thrombus in 17 cases (48.57%) (17/35), pericatheteric sheath was the next most common, with 12 cases (34.29%), and mixed thrombus with 4 cases (11.43%), in addition to pulmonary embolism in 2 cases (5.71%) and the common site of thrombosis was internal jugular vein thrombosis. Symptomatic thrombi occurred in 31 cases (88.57%), with the main presenting symptom being the development of localized pain or infection. The demographic data, disease, laboratory data and venous access-related data of both groups are shown in **Table 1**. There were no differences between the two sets regarding the Age, Gender, Diagnosis, Activity amount, Surgical history, KPS score, BMI, smoking history drinking history, Hypertension, Diabetes mellitus, Hyperlipidemia, Thrombosis history(within the last 12 months), Catheter type, History of central venous catheterization, Catheter side, Punctured vein, and Position of catheter tip (all *P*>0.05). In **Table 2** we present the demographic and clinicopathological characteristics of patients in the training set, in this study, ROC curve is used to find the cut-off value of the laboratory indicators.

**Table 1:** Demographic and clinical characteristics among all patients.

Characteristic	Entire cohort (n=305) No. (%)	Training set (n=215) No. (%)	Validation set (n=90) No. (%)	*P*
**Age (years, x ± s)**	**52.13 ± 14.94**	**52.43 ± 15.39**	**51.43 ± 13.89**	**0.597**
**Gender**				**0.221**
**Male**	**185 (60.66)**	**126 (58.6)**	**59 (65.6)**	
**Female**	**120 (30.34)**	**89 (41.4)**	**31 (34.4)**	
**Diagnosis**				**0.582**
**Non-Hodgkin’s lymphoma (NHL)**	**246 (80.7)**	**175 (81.4)**	**71 (78.9)**	
**Hodgkin’s lymphoma (HL)**	**59 (19.3)**	**40 (18.6)**	**19 (21.1)**	
Activity amount*				**0.322**
**Hardly**	**22 (7.2)**	**15 (7.0)**	**7 (7.8)**	
**Frequently**	**283 (92.8)**	**200 (93.0)**	**83 (92.2)**	
**Surgical history (within the last 6 months)**				**0.756**
**No**	**186 (61.0)**	**130 (60.5)**	**56 (62.2)**	
**Yes**	**119 (39.0)**	**85 (39.5)**	**34 (37.8)**	
**PICC history of blood transfusion (within the last 6 months)**				**0.012**
**No**	**282 (92.4)**	**194 (90.2)**	**88 (97.8)**	
**Yes**	**23 (7.6)**	**21 (9.8)**	**2 (2.2)**	
**KPS score**				**0.452**
**≥90points**	**184 (60.3)**	**133 (61.9)**	**51 (56.7)**	
**≤80 points**	**121 (39.7)**	**82 (38.1)**	**39 (43.3)**	
**BMI (kg/m^2^)**				**0.349**
**≤25**	**252 (82.6)**	**175 (81.4)**	**77 (85.6)**	
**>25**	**53 (17.4)**	**40 (18.6)**	**13 (14.4)**	
**smoking history**				**0.937**
**No**	**216 (70.8)**	**152 (70.7)**	**64 (71.1)**	
**Yes**	**89 (29.2)**	**63 (29.3)**	**26 (28.9)**	
**drinking history**				**0.231**
**No**	**258 (84.6)**	**185 (86.0)**	**73 (81.1)**	
**Yes**	**47 (15.4)**	**30 (14.0)**	**17 (18.9)**	
**Co-infection**				**0.048**
**No**	**229 (75.1)**	**173 (80.5)**	**56 (62.2)**	
**Yes**	**76 (24.9)**	**42 (19.5)**	**34 (37.8)**	
**Hormone use**				**<0.001**
**No**	**43 (14.1)**	**42 (19.5)**	**1 (1.1)**	
**Yes**	**262 (85.9)**	**173 (80.5)**	**89 (98.9)**	
**Hypertension**				**0.453**
**No**	**272 (89.2)**	**190 (88.4)**	**82 (91.1)**	
**Yes**	**33 (10.8)**	**25 (11.6)**	**8 (8.9)**	
**Diabetes mellitus**				**0.746**
**No**	**293 (96.1)**	**207 (96.3)**	**86 (95.6)**	
**Yes**	**12 (3.9)**	**8 (3.7)**	**4 (4.4)**	
**Hyperlipidemia**				**0.174**
**No**	**221 (72.5)**	**175 (81.4)**	**46 (51.5)**	
**Yes**	**84 (27.5)**	**40 (18.6)**	**44 (48.9)**	
**Thrombosis history (within the last 12 months)**				**0.107**
**No**	**279 (91.5)**	**201 (93.5)**	**78 (86.7)**	
**Yes**	**26 (8.5)**	**14 (6.5)**	**12 (13.3)**	
**Catheter type **				**0.840**
**4Fr**	**194 (63.6)**	**177 (82.3)**	**73 (81.1)**	
**5Fr**	**111 (36.4)**	**38 (17.7)**	**17 (18.9)**	
**History of central venous catheterization**				**0.434**
**yes**	**182 (59.7)**	**124 (57.7)**	**58 (64.4)**	
**no**	**123 (40.3)**	**91 (42.3)**	**32 (35.6)**	
**Catheter side **				**0.505**
**Left**	**260 (85.2)**	**185 (86.0)**	**75 (83.3)**	
**Right**	**45 (14.8)**	**30 (14.0)**	**15 (16.7)**	
**Punctured vein **				**0.094**
**Precious Veins**	**276 (90.5)**	**200 (93.0)**	**76 (84.4)**	
**Brachial vein**	**22 (7.2)**	**13 (6.0)**	**9 (10.0)**	
**Cephalic vein **	**26 (2.3)**	**2 (1.0)**	**5 (5.6)**	
**Position of catheter tip **				**0.824**
**Upper half of superior vena cava**	**19 (6.2)**	**13 (6.0)**	**6 (6.7)**	
**Lower half of superior vena cava**	**286 (93.8)**	**202 (94.0)**	**84 (93.3)**	

Activity amount*:Change in daily activity at the site of placement compared to activity before placement. Hardly: Significant reduction in daily activity at the site of placement compared to pre-tubing activity. Frequently: Daily activity at the site of placement is unchanged or increased compared to pre-tubing activity. Bold values: KPS score, Karnofsky Performance Status score.

**Table 2:** Demographic and clinicopathological characteristics of patients in the training set.

Characteristic	Non-PICC-RVTE (n = 194)	PICC-RVTE (n = 21)	x²	P
Demographic characteristics				
**Age (years)**			**1.938**	**0.126**
**≤60**	**131 (67.5)**	**11 (52.4)**		
**>60**	**63 (32.5)**	**10 (47.6)**		
**Gender**			**0.104**	**0.469**
**Male**	**113 (58.2)**	**13 (61.9)**		
**Female**	**81 (41.8)**	**8 (38.1)**		
**Diagnosis**			**2.945**	**0.067**
**Non-Hodgkin’s ** ** lymphoma (NHL)**	**155 (79.9)**	**20 (95.2)**		
**Hodgkin’s ** ** lymphoma (HL)**	**39 (20.1)**	**1 (4.8)**		
**Activity amount**			**10.161**	**0.009**
**Hardly**	**10 (5.2)**	**5 (23.8)**		
**Frequently**	**184 (94.8)**	**16 (76.2)**		
**Surgical history (within the last 6 months)**			**0.107**	**0.458**
**No**	**118 (60.8)**	**12 (57.1)**		
**Yes**	**76 (39.2)**	**9 (42.9)**		
**PICC history of blood transfusion (within the last 6 months)**			**9.337**	**0.009**
**No**	**179 (92.3)**	**15 (71.4)**		
**Yes**	**15 (7.7)**	**6 (28.6)**		
**KPS score**			**5.098**	**0.023**
**≥90points**	**123 (63.4)**	**8 (38.1)**		
**≤80 points**	**71 (36.6)**	**13 (61.9)**		
**BMI (kg/m^2^)**			**0.287**	**0.425**
**≤25**	**157 (80.9)**	**18 (85.7)**		
**>25**	**37 (19.1)**	**40 (14.3)**		
**smoking history**			**2.064**	**0.120**
**No**	**140 (72.2)**	**12 (57.1)**		
**Yes**	**54 (27.8)**	**9 (42.9)**		
**drinking history**			**4.142**	**0.052**
**No**	**170 (87.6)**	**15 (71.4)**		
**Yes**	**24 (12.4)**	**6 (28.6)**		
**Co-infection**			**8.053**	**0.009**
**No**	**161 (83.0)**	**12 (57.1)**		
**Yes**	**33 (17.0)**	**9 (42.9)**		
**Hormone use**			**0.408**	**0.381**
**No**	**39 (20.1)**	**3 (14.3)**		
**Yes**	**155 (79.9)**	**18 (85.7)**		
**Hypertension**			**0.100**	**0.547**
**No**	**171 (88.1)**	**19 (90.5)**		
**Yes**	**23 (11.9)**	**2 (9.5)**		
**Diabetes mellitus**			**0.070**	**0.567**
**No**	**187 (96.4)**	**20 (95.2)**		
**Yes**	**7 (3.6)**	**1 (4.8)**		
**Hyperlipidemia**			**3.334**	**0.069**
**No**	**161 (83.0)**	**14 (66.7)**		
**Yes**	**33 (17.0)**	**7 (33.3)**		
**Thrombosis history (within the last 12 months)**			**18.604**	**0.001**
**No**	**186 (95.9)**	**15 (71.4)**		
**Yes**	**8 (4.1)**	**6 (28.6)**		
Laboratory data				
**RBC (10^12^/l)**			**1.938**	**0.124**
** ≤4.0**	**63 (32.5)**	**10 (47.6)**		
** >4.0**	**131 (67.5)**	**11 (52.4)**		
WBC* (10^9^/l)			**0.770**	**0.680**
**<4**	**47 (24.2)**	**6 (28.6)**		
**4-10**	**120 (61.9)**	**11 (52.4)**		
**>10**	**27 (13.9)**	**4 (19.0)**		
**PLT (10^9^/l)**			**0.183**	**0.913**
**<100**	**22 (11.3)**	**3 (14.3)**		
**100-300**	**131 (67.5)**	**14 (66.7)**		
**>300**	**41 (21.1)**	**4 (19.0)**		
**HGB (g/l)**			**0.282**	**0.381**
**≤110**	**140 (72.2)**	**14 (66.7)**		
**>110**	**54 (27.8)**	**7 (33.3)**		
**HCT**			**1.804**	**0.133**
**≤40**	**109 (56.2)**	**15 (71.4)**		
**>40**	**85 (43.8)**	**6 (28.6)**		
**PT (s)**			**0.081**	**0.481**
**≤12.1**	**41 (21.1)**	**5 (23.8)**		
**>12.1**	**153 (78.9)**	**16 (76.2)**		
**INR**			**0.068**	**0.543**
**≤1.06**	**162 (83.5)**	**18 (85.7)**		
**>1.06**	**32 (16.5)**	**3 (14.3)**		
**APTT (s)**			**2.872**	**0.082**
**≤39.6**	**152 (78.4)**	**13 (61.9)**		
**>39.6**	**42 (21.6)**	**8 (38.1)**		
**TT**			**0.442**	**0.336**
**≤15**	**43 (22.2)**	**6 (28.6)**		
**>15**	**151 (77.8)**	**15 (71.4)**		
**FIB**			**7.004**	**0.008**
**≤4**	**114 (58.8)**	**6 (28.6)**		
**>4**	**80 (41.2)**	**15 (71.4)**		
**ATIII**			**6.339**	**0.013**
**≤97.5**	**66 (34.0)**	**13 (61.9)**		
**>97.5**	**128 (66.0)**	**8 (38.1)**		
**FDP**			**2.084**	**0.121**
**<5**	**148 (76.3)**	**13 (61.9)**		
**≥5**	**46 (23.7)**	**8 (38.1)**		
**D-Dimer (mg/L)**				
**≤0.55**	**102 (52.9)**	**4 (19.0)**	**8.523**	**0.003**
**>0.55**	**92 (47.4)**	**17 (81.0)**		
**Albumin (g/L)**			**4.594**	**0.040**
**≤35.5**	**29 (14.9)**	**7 (33.3)**		
**>35.5**	**165 (85.1)**	**14 (66.7)**		
**Albumin/Globulin**			**1.357**	**0.178**
**≤1.62**	**59 (30.4)**	**9 (42.9)**		
**>1.62**	**135 (69.6)**	**12 (57.1)**		
**Total bilirubin (μmol/L)**			**1.981**	**0.119**
**≤9.05**	**96 (49.5)**	**7 (33.3)**		
**>9.05**	**98 (50.5)**	**14 (66.7)**		
**Total cholesterol (mmol/L)**			**3.895**	**0.048**
**<5.36**	**149 (76.8)**	**12 (57.1)**		
**≥5.36**	**45 (23.2)**	**9 (42.9)**		
**Fasting Blood Glucose (mmol/l)**			**1.171**	**0.244**
**<6.11**	**169 (87.1)**	**20 (95.2)**		
**≥6.11**	**25 (12.9)**	**1 (4.8)**		
Indicators related to PICC placement				
**Catheter type n (%)**			**0.030**	**0.529**
**4Fr**	**160 (82.5)**	**17 (81.0)**		
**5Fr**	**34 (17.5)**	**4 (19.0)**		
**History of central venous catheterization**			**0.771**	**0.262**
**yes**	**110 (56.7)**	**14 (66.7)**		
**no**	**84 (43.3)**	**7 (33.3)**		
**Catheter side n (%)**			**0.503**	**0.333**
**Left**	**168 (86.6)**	**17 (81.0)**		
**Right**	**26 (13.4)**	**4 (19.0)**		
**Punctured vein n (%)**			**4.272**	**0.118**
**Precious Veins**	**182 (93.8)**	**18 (85.7)**		
**Brachial vein**	**11 (5.7)**	**2 (9.5)**		
**Cephalic vein**	**1 (0.5)**	**1 (4.8)**		
**Position of catheter tip n (%)**			**30.503**	**<0.001**
**Upper half of superior vena cava**	**6 (3.1)**	**7 (33.3)**		
**Lower half of superior vena cava**	**188 (96.9)**	**14 (66.7)**		

KPS score, Karnofsky Performance Status score; BMI, Body Mass Index; RBC, Red Blood Cell; WBC, White Blood Cell; PLT, Platelet; HGB, Hemoglobin; HCT, Hematocrit; PT, Prothrombin Time; INR, International Normalized Ratio; APTT, Activated Partial; Thromboplastin Time; TT, Thrombin Time; FIB, Fibrinogen; ATIII, Antithrombin III; FDP, Fibrinogen Degradation Products.

### 3.2 The potential risks and multivariate analysis

**Table 3** presents the potential risks of PICC associated thrombosis in 215 analyzed by univariate logistic regression analysis. There were statistically significant differences in Activity amount (OR: 0.174; 95% CI:0.053-0.571; *P*=0.004), KPS score (OR: 2.815; 95% CI: 1.113-7.120; *P*=0.029), drinking history (OR: 2.833; 95% CI: 1.003-8.006; P=0.049), PICC history of blood transfusion (within the last 6 months) (OR: 4.773; 95% CI: 1.615-14.105; *P*=0.005), Co-infection (OR: 3.695; 95% CI: 1.427-9.385; *P*=0.007), Thrombosis history(within the last 12 months) (OR: 9.300; 95% CI: 2.852-30.327;*P*<0.001), FIB (OR: 3.562; 95% CI: 1.325-9.578; *P*=0.012), ATIII(within the last 12 months) (OR: 0.317; 95% CI: 0.125-0.804;P=0.015), Albumin (OR: 0.352; 95% CI: 0.131-0.945; P=0.038), Total cholesterol (OR: 2.4837; 95% CI: 0.983-6.271;P=0.035), Position of catheter tip(OR: 0.064; 95% CI:0.019-0.216;P<0.001, and D-dimer (OR: 4.712; 95% CI: 1.530-14.515).

**Table 3:** Univariate logistic regression analysis in the training set.

Characteristics	OR (95%CI)	*P*
Activity amount		
**Hardly**	**Reference**	
**Frequently**	**0.174 (0.053-0.571)**	**0.004**
KPS score		
**≥90points**	**Reference**	
**≤80 points**	2.815 **(1.113-**7.120)	**0.029**
drinking history		
**No**	**Reference**	
**Yes**	**2.833 (1.003-8.006)**	**0.049**
PICC history of blood transfusion (within the last 6 months)		
**No**	**Reference**	
**Yes**	**4.773 (1.615-14.105)**	**0.005**
Co-infection		
**No**	**Reference**	
**Yes**	**3.659 (1.427-9.385)**	**0.007**
Thrombosis history (within the last 12 months)		
**No**	**Reference**	
**Yes**	**9.300 (2.852-**30.327)	**<0.001**
FIB		
**≤4**	**Reference**	
**>4**	**3.562 (1.325-9.578)**	**0.012**
ATIII		
**≤97.5**	**Reference**	
**>97.5**	**0.317 (0.125-0.804)**	**0.015**
D-Dimer (mg/L)		
**≤0.55**	**Reference**	
**>0.55**	**4.712 (1.530-14.515)**	**0.007**
Albumin (g/L)		
**≤35.5**	**Reference**	
**>35.5**	**0.352 (0.131-0.945)**	**0.038**
Total cholesterol (mmol/L)		
**<5.36**	**Reference**	
**≥5.36**	**2.483 (0.983-6.271)**	**0.035**
Position of catheter tip		
**Upper half of superior vena cava**	**Reference**	
**Lower half of superior vena cava**	**0.064 (0.019-0.216)**	**<0.001**

KPS score, Karnofsky Performance Status score; FIB, Fibrinogen; ATIII, Antithrombin III.

Independent factors associated with PICC-RVTE were further analyzed by multivariate logistic regression, and the results are presented in **Table 4**. It was found that Activity amount, Thrombosis history(within the last 12 months), ATIII, Total cholesterol, Position of catheter tip, and D-dimer levels were the main influencing factors of catheter thrombosis (*P*<0.05).

**Table 4:** Multivariable logistic regression analysis in the training set.

Characteristics	OR (95%CI)	*P*
Activity amount		
**Hardly**	**Reference**	
**Frequently**	**0.217 (0.045-1.038)**	**0.006**
Thrombosis history (within the last 12 months)		
**No**	**Reference**	
**Yes**	**33.733 (5.964-190.806)**	**<0.001**
ATIII		
**≤97.5**	**Reference**	
**>97.5**	**0.182 (0.052-0.634)**	**0.007**
Total cholesterol (mmol/L)		
**<5.36**	**Reference**	
**≥5.36**	**3.865 (1.087-13.744)**	**0.037**
Position of catheter tip n (%)		
**No**	**Reference**	
**Yes**	**0.057 (0.012-0.282)**	**<0.001**
D-Dimer (mg/L)		
**≤0.55**	**Reference**	
**>0.55**	**5.765 (1.205-25.578)**	**0.028**

ATIII, Antithrombin III.

### 3.3 Nomogram for PICC-RVTE prediction and validation

**Figure 3:**
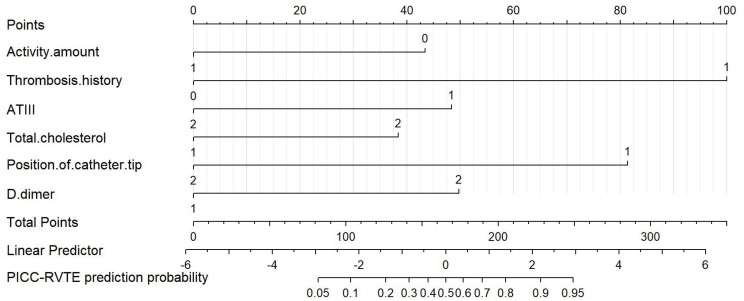
Nomogram for predicting PICC-RVTE risk in lymphoma patients after PICC catheterization. To use the nomogram, the points corresponding to each prediction variable were obtained, then the sum of the points was calculated as the total score, and the predicted risk corresponding to the total score was the probability of PICC-RVTE.

**Figure 4:**
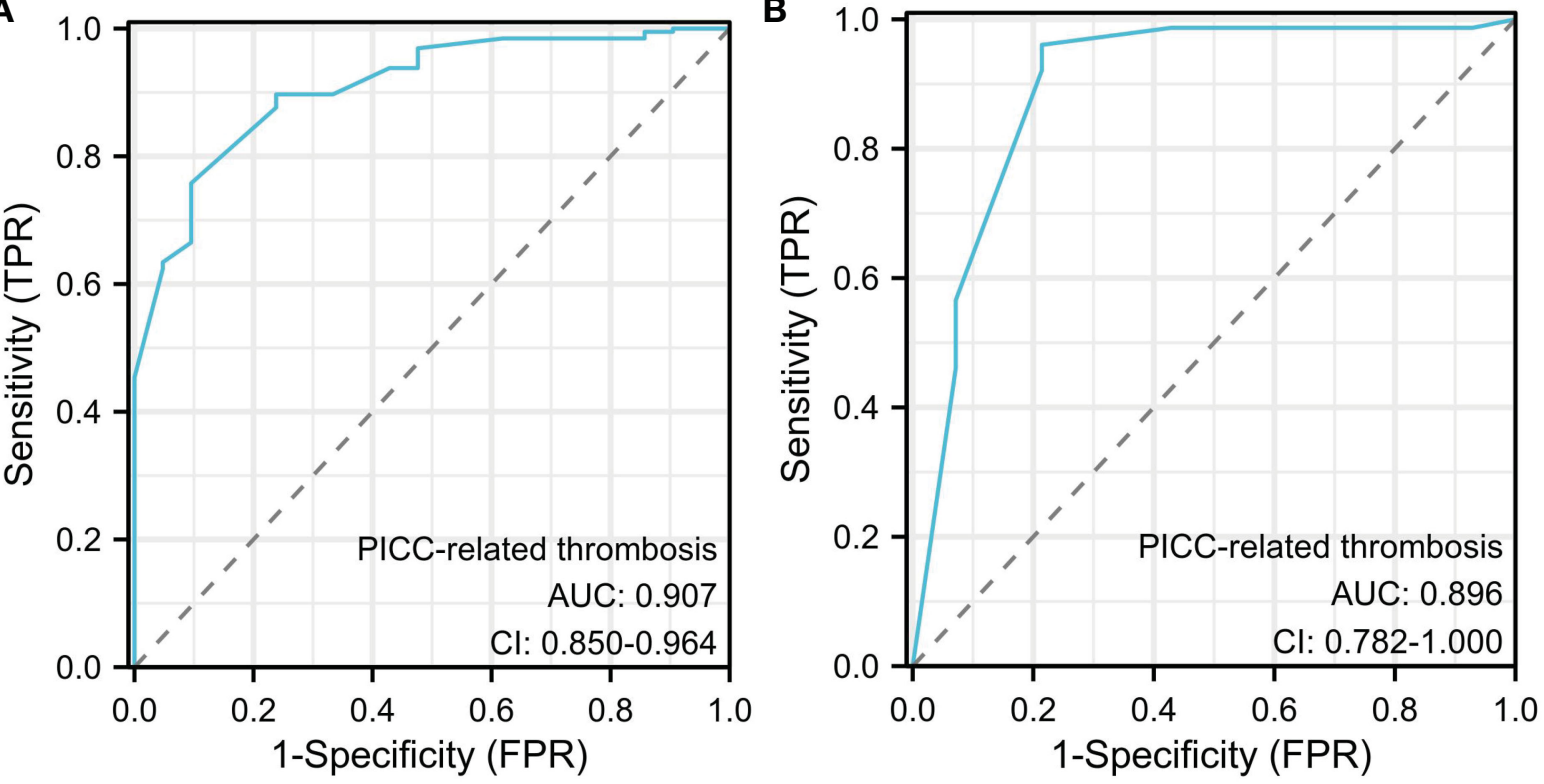
Receiver operating characteristic (ROC) curve analysis for PICC-RVTE risk prediction. ROC curves of PICC-RVTE risk prediction in the training set **(A)** and the testing set **(B)**. AUC was calculated using bootstrapping, and its 95% CI was estimated. The P-value were two-sided. The AUC and 95% CI in the training set and the testing set were 0.907(95%CI:0.850-0.964) and 0.896(95%CI: 0.782-1.000), respectively, and Delong test P>0.05.

**Figure 5:**
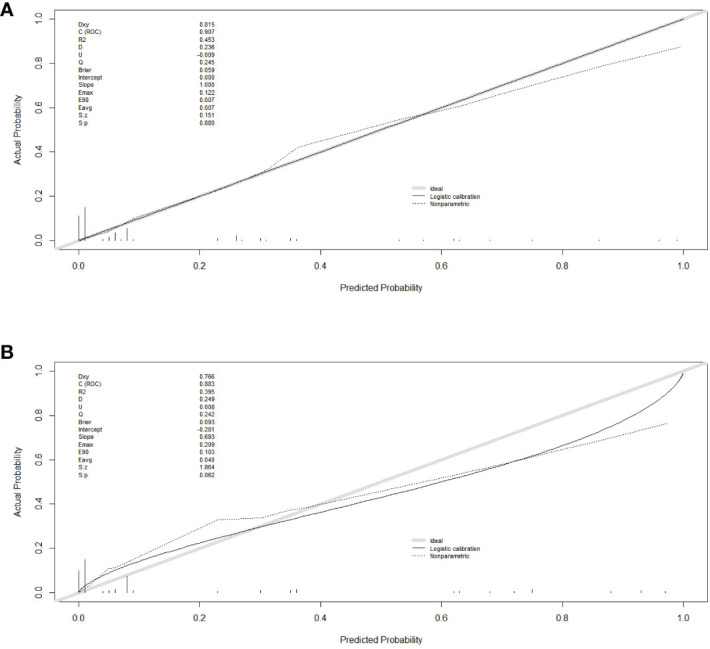
Calibration curves in training and validation sets. **(A)** calibration curve in the training cohort; **(B)** calibration curve in the validation cohort. The gray thick line represents a perfect prediction by an ideal model, the black dashed line indicates the target parameter and the solid black line shows the performance of the model. Using bootstrap resampling (times = 1000).

**Figure 6:**
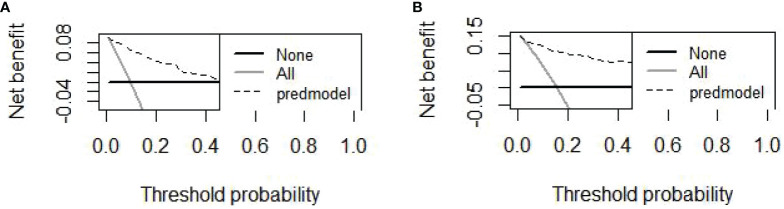
The DCAs curve of the nomogram was observed in both the training and validation cohorts. **(A)** The decision curve of the nomogram for predicting PICC-RVTE risk in the training cohort; **(B)** The decision curve of the nomogram for predicting PICC-RVTE risk in the validation cohort. The prediction model is represented by a black dashed line, while the gray solid line represents all samples that were intervened, and the black solid horizontal line represents all samples that were not intervened. The graph illustrates the expected net benefit of each patient in relation to the nomogram’s ability to predict PICC-RVTE formation risk. The net benefit increases as the model curve extends.

Finally, six independent factors were included in the construction of the nomogram for patients with lymphoma after PICC implantation (**Figure 3**). The C-index of the nomogram forecasting model was 0.907 (95%CI:0.850-0.964), and the model had good accuracy. The external validation cohort (n =90) showed an accuracy area under the curve(AUC) (**Figures 4A, B**) of 0.896(95%CI: 0.782-1.000).The calibration curves (**Figures 5A, B**) showed good agreement between the predicted probabilities and the actual observations of the obtained response prediction model for the training cohort and external validation cohort. The decision curve analysis (**Figures 6A, B**) lies above both the None and All lines, quantitatively showing that the model has clinical utility.

## 4 Discussion

At present, the application of PICC in oncology inpatient wards is very common. Compared with central venous catheterization (CVC), PICC has the advantages of low price, avoiding repeated puncture, relatively simple operation and nursing care, convenient clinical drug administration, not affecting normal activities, and providing long-term and stable vascular access for chemotherapeutic drugs, parenteral nutrition, and hemodynamic monitoring, etc., which is now gradually being preferred by the majority of healthcare professionals in clinical application (6, 7). However, the regular utilization of the peripherally inserted central catheter (PICC) on a weekly basis renders it vulnerable to bloodstream infection and the development of PICC-RVTE. Consequently, patients are frequently subjected to the necessity of undergoing weekly dressing changes, thereby increasing the incidence of infection and the occurrence of PICC-RVTE formation as prevalent complications (8). PICC-related infection is a serious complication that may lead to prolonged hospitalization, increased healthcare costs, and even death (9). Currently, there are many studies on PICC-RVTE, but we are the first to investigate the PICC-RVTE in lymphoma patients with the basic characteristics and major risk factors, culminating in a highly sensitive and specific clinical prediction model to prevent PICC-RVTE and improve the management of PICC-RVTE.

### 4.1 Analysis of PICC-RVTE formation

The wide variability in reported incidences of PICC-related venous thromboembolism (PICC-RVTE), ranging from 3.0% to 75.0%, can be attributed to factors such as diverse study populations, diagnostic methods, and the attention paid by clinical practitioners to asymptomatic thrombosis10, 11. In our study involving 305 lymphoma patients with PICC, we observed a PICC-RVTE incidence of 11.48% (35/305), significantly higher than the reported 3%. The main reason may be that both symptomatic and asymptomatic patients with thrombosis in this study were validly included in the experiment and analyze, as well as the recognition of tumor-associated thrombosis as a significant concern by the medical personnel in our research department prompted the implementation of frequent vascular color Doppler ultrasonography for all hospitalized patients. Consequently, we were able to identify thrombosis prior to the manifestation of symptoms, resulting in a higher observed prevalence of thrombosis compared to previous reports. Importantly, we found symptomatic thrombosis presenting as predominantly localized pain and infection in 31 cases (88.57%), which is similar to the current report (10, 11). It is generally accepted that the longer a PICC catheter is retained, the greater the risk of catheter-related thrombosis, our previous study, which examined CVC placement in patients with malignancy, revealed that the average time interval between CVC placement and the formation of catheter-related thrombosis was 10.01 ± 6.053 days. In our current analysis of the PICC in lymphoma patients, we found that the mean duration of thrombosis from PICC insertion to thrombosis was 13.00 days. This final finding aligns with previous literature reports (12, 13), further emphasizing the importance of preventive measures within the first two weeks following PICC insertion. In addition, we also analyzed three types of thrombus, vascular adherent thrombus, pericatheteric sheath, and mixed thrombus, according to the site of catheter-related thrombosis. Our team found that vascular adherent thrombus occurring in the internal jugular vein was the most common PICC catheter-associated thrombus, and a review of the relevant literature has not yet resulted in similar reports.

### 4.2 Risk factor analysis, nomogram prediction modeling and model discrimination, calibration and evaluation of clinical applicability for PICC-RVTE

In recent studies, numerous and controversial risk factors for PICC-RVTE were reported (14). Pathophysiologically, intimal injury or inflammation of the vein, slow blood flow, and blood hypercoagulability are the three main causes of thrombosis (15). Based on our study’s results, the most significant complication is pulmonary embolism caused by dislodging venous thrombi, which resulted in death.Hence, a crucial component of our study involved the analysis of risk factors associated with the formation of PICC-RVTE, as well as the implementation of preventive measures against PICC-RVTE formation. The risk factors for catheter-related thrombosis exhibit significant variation across previous studies. However, a more traditional perspective categorizes these risk factors into three types: placement factors (e.g., catheter material, site of placement, location of catheter tip, and number of catheter punctures), patient-related factors (e.g., age, comorbidities, history of surgery, history of venous thromboembolism, and advanced age), and therapeutic factors (e.g., the utilization of chemotherapy, transfusions, and prophylactic anticoagulants) (12, 16, 17). Incorporating a more extensive examination of risk factors, our study focused on lymphoma patients who underwent PICC placement, leading to the identification of several key determinants of catheter thrombosis, namely activity level, thrombosis history within the past 12 months, ATIII levels, total cholesterol levels, and D-dimer levels. There is no doubt that less activity level is one of the most significant risk factors for thrombosis (18, 19). The primary causative factor behind this phenomenon is the deceleration of regional blood circulation resulting from the diminished muscular pumping capacity of the cannulated limb. This, in turn, leads to venous dilation, endothelial impairment, and the accumulation of coagulation factors that trigger the activation of the coagulation system, ultimately facilitating the development of thrombus. Consequently, numerous scholarly works have emphasized the significance of health education pertaining to mechanical prophylaxis, including healthcare practitioners advising patients to engage in upper limb relaxation and clenching at the site of peripheral central venous catheter insertion as a preventive measure against the formation of PICC-RVTE (20, 21). Furthermore, our study specifically examined the association between a history of thrombosis within the previous 12 months and the formation of PICC-RVTE. Our findings revealed a significantly high odds ratio of 33.733 (95% confidence interval, 5.964-190.806), which aligns with previous research conducted by Yang et al. (10), Marin et al. (22), Guy et al. (23), and other research teams. This emphasizes the importance of providing special clinical attention to patients with malignant tumors who have a history of thrombosis. D-dimer (D-D) serves as a recognized marker for assessing coagulation and fibrinolytic activity, enabling an indirect determination of thrombotic activity and serving as a reliable indicator for evaluating the extent of coagulation system activation (24). The medical community now widely acknowledges the prevalence of elevated plasma D-D levels in patients with tumors, a phenomenon that appears to be closely associated with the heightened thrombotic risk observed in individuals with malignant tumors. On the other hand, antithrombin III (ATIII) represents a crucial inhibitor of coagulation factors, and even slight alterations in ATIII levels can significantly impact the risk of thromboembolism (25). Our analysis of coagulation mechanisms revealed that D-D and ATIII serve as significant indicators of thrombotic complications and added the importance of coagulation laboratory tests, aligning with the findings of the present study (10). The elevated occurrence of elevated plasma cholesterol represents a significant health concern in Europe (26). Extensive research has been conducted on the association between hyperlipidemia, atherosclerosis, and thrombosis (27, 28). It has long been documented that hypercholesterolemia contributes to heightened platelet aggregation (29). In our study, we have incorporated high plasma cholesterol as a laboratory indicator, and the findings indicate its correlation with catheter-related thrombosis. This observation can be attributed to the association between hyperlipidemia and oxidative stress, the production of oxidized lipoproteins, and an elevated susceptibility to thrombosis. Consequently, this significant discovery underscores the imperative of actively managing hypertension, hyperlipidemia, and other conventional risk factors associated with thrombosis. The formation of PICC-RVTE is influenced by various factors, including catheter-related factors such as operator proficiency and experience, puncture duration and frequency, catheter gauge, venous valves and anatomical variations, regular maintenance, and Patient bedridden time. Typically, a single peripherally inserted central catheter (PICC) is positioned in the lower one-third of the junction between the superior vena cava (SVC) and caval atrium. Recent studies (3, 30, 31) have provided evidence that catheter-related thrombosis is linked to catheter displacement. Moreover, the occurrence of venous thrombosis associated with peripherally inserted central catheters (PICCs) is notably higher when the catheter tip is positioned at the superior end of the superior vena cava. In order to mitigate the risk of thrombosis resulting from catheter displacement, it is recommended that the catheter tip be placed in the inferior segment of the superior vena cava. This positioning should be confirmed through real-time electrocardiographic monitoring and verified by postoperative X-ray, rather than relying on transesophageal ultrasound. In this study, the identified PICC-RVTE occurred in catheter tip migration after PICC.

Surprisingly, our study did not find any significant associations between recognized risk factors (3, 5, 15) such as age, history of surgery, history of smoking, hypertension, diabetes mellitus, and selection of puncture veins with PICC-related venous thrombosis. This discrepancy may be attributed to variations in sample selection within our study population, as well as limitations and selection bias in the two volumes of data. Additionally, the limited information available on the covariates of interest and the failure to separately analyze patients with asymptomatic thrombosis in our study could have contributed to these findings.

As previously stated, we have successfully incorporated significant risk factors into our final analysis through a comprehensive examination of these factors. Consequently, our risk prediction model has demonstrated commendable discriminatory and calibration abilities across both dual-center study centers. Furthermore, the reliability and enhanced clinical benefit potential of our model have been substantiated. Notably, this marks the first instance of a clinical prediction model being established for the prediction of PICC-related venous thromboembolism in lymphoma patients subsequent to PICC placement, as far as our knowledge extends.

### 4.3 Study advantages and limitations

Notwithstanding the aforementioned advantages, our study is subject to certain limitations. Firstly, the sample size of the study, derived from two medical centers, was relatively small, potentially impeding the identification of an adequate number of potential risk factors. Secondly, the retrospective design of this study restricted the analysis to a limited number of factors, such as operator experience, patients’ individual treatment, and the intricate and diverse nature of catheterization factors, which may exert a substantial influence on PICC-RVTE. Additionally, the limited sample size may have hindered the detection of significant differences between groups, thereby compromising the efficacy of our study. As a result of the constraints imposed by our sample selection and the presence of missing data, certain factors known to exert significant influence on PICC-RVTE, including stage, International Prognostic Index (IPI), germinal center, and active B cells, antitumor therapy (32) were not incorporated into subsequent analyses. This omission underscores the need for further exploration in future prospective studies with larger sample sizes.

## 5 Conclusion

This study examines the clinical characteristics of PICC-related upper extremity deep vein thrombosis (PICC-RVTE) in lymphoma patients, identifies associated risk factors, underscores the significance of early detection within the initial two weeks following PICC implantation, and ultimately establishes and validates a novel predictive model for assessing the risk of PICC-RVTE in cancer patients. This user-friendly model chart holds potential as a valuable tool in clinical practice. This straightforward model effectively forecasts the likelihood of PICC-RVTE incidence, and its application can assist healthcare professionals in making informed choices regarding prevention strategies in cancer patients, as well as offering insights for timely monitoring and identification of thrombotic occurrences.

## Data availability statement

The original contributions presented in the study are included in the article/supplementary material. Further inquiries can be directed to the corresponding author.

## Ethics statement

The studies involving humans were approved by the Medical Research Ethics Committee of Yunnan Cancer Hospital and the First People’s Hospital of Anning, which is affiliated with Kunming University of Science and Technology (Approval NO.2023-018-01 & No. KYLX2023-101). The studies were conducted in accordance with the local legislation and institutional requirements. The ethics committee/institutional review board waived the requirement of written informed consent for participation from the participants or the participants’ legal guardians/next of kin because of the retrospective nature of this clinical investigation, and given that all patient data were de-identified.

## Author contributions

XW: Writing – original draft. YH: Data curation, Writing – review & editing. JC: Writing – review & editing, Formal analysis. JX: Writing – review & editing.
